# Addition of Cryoprotectant DMSO Reduces Damage to Spermatozoa of Yellow Catfish (*Pelteobagrus fulvidraco*) during Cryopreservation: Ultrastructural Damage, Oxidative Damage and DNA Damage

**DOI:** 10.3390/ani14182652

**Published:** 2024-09-12

**Authors:** Yuxin Zhang, Dongqing Liu, Qinghua Wang, Qingxin Ruan, Sijie Hua, Weiwei Zhang, Sen Yang, Zining Meng

**Affiliations:** 1 State Key Laboratory of Biocontrol, Southern Marine Science and Engineering Guangdong Laboratory (Zhuhai), Institute of Aquatic Economic Animals and Guangdong Provincial Key Laboratory of Aquatic Economic Animals, School of Life Sciences, Sun Yat-sen University, Guangzhou 510275, China; zhangyx558@mail2.sysu.edu.cn (Y.Z.); sheepskin163@163.com (D.L.); wangqh55@mail2.sysu.edu.cn (Q.W.); ruanqx3@mail2.sysu.edu.cn (Q.R.); huasj@mail2.sysu.edu.cn (S.H.); zhangww79@mail2.sysu.edu.cn (W.Z.); 2 College of Food Science and Technology, Guangdong Ocean University (Yangjiang Campus), Yangjiang 529599, China; yangsen@gdou.edu.cn; 3 China-ASEAN Belt and Road Joint Laboratory on Mariculture Technology, Guangzhou 510275, China

**Keywords:** yellow catfish, spermatozoa cryopreservation, motility, DMSO, ultrastructure, oxidative damage, DNA integrity

## Abstract

**Simple Summary:**

Spermatozoa cryopreservation technology has been widely used to preserve high-quality male spermatozoa and has to some extent solved the problem of spermatozoa supply in yellow catfish (*Pelteobagrus fulvidraco*). However, cryopreservation can still cause cellular damage and affect spermatozoa viability and fertility. Therefore, it is necessary to study cryo-injury, which is beneficial for further optimizing the spermatozoa cryopreservation protocol. In this experiment, we investigated the effects of adding or not adding 10% DMSO on spermatozoa motility, ultrastructural damage, oxidative damage, and DNA damage of thawed yellow catfish. Using 10% DMSO as a cryoprotectant can improve spermatozoa motility (75.64 ± 1.12), significantly alleviate injuries to organelles and membrane structures, improve SOD (7.99 × 10^4^ ± 5.24 nmol/mgprot) and T-AOC (3.7 × 10^5^ ± 0.06 nmol/mgprot) enzyme activity, reduce MDA (1.09 ± 0.45 nmol/mgprot) enzyme activity, and mitigate the degree of oxidative damage. It also greatly reduces the DNA fragmentation rate (59 ± 1.56%). The research results provide data support for the optimization of the cryopreservation protocol for the spermatozoa of yellow catfish, which is beneficial for the preservation of the germplasm resources of yellow catfish and the effective utilization of resources during large-scale production.

**Abstract:**

Spermatozoa cryopreservation protocols have been established for yellow catfish (*Pelteobagrus fulvidraco*), but cryopreservation can still cause cellular damage and affect spermatozoa viability and fertility. Therefore, the aim of this paper was to evaluate the effects of adding or not adding cryoprotectants during low-temperature storage on the ultrastructural damage, oxidative damage, and DNA damage of thawed yellow catfish spermatozoa. The mixed semen of three male yellow catfish was divided into a fresh spermatozoa group, a frozen spermatozoa group (DMSO^+^) with a cryoprotectant (10% DMSO), and a frozen spermatozoa group without a cryoprotectant (DMSO^−^). Ultrastructural of the spermatozoa after thawing were observed under an electron microscope and the spermatozoa were assayed for SOD, MDA, and T-AOC enzyme activities, as well as for DNA integrity. In terms of movement parameters, compared with DMSO^−^, the addition of DMSO has significantly improved sperm motility, curve line velocity (VCL), and straight line velocity (VSL). The ultrastructural results showed that although thawed spermatozoa exhibited increased damage than fresh spermatozoa, 10% DMSO effectively reduced the damage to the plasma membrane, mitochondria, and flagellum of spermatozoa by cryopreservation, and most of the spermatozoa were preserved with intact structure. The results of oxidative damage showed that compared with frozen spermatozoa, 10% DMSO significantly increased the activities of SOD and T-AOC enzymes and clearly reduced the activity of the MDA enzyme. The antioxidant capacity of spermatozoa was improved, lipid peroxidation was reduced, and the oxidative damage caused by cryopreservation was mitigated. The DNA integrity of spermatozoa showed that 10% DMSO clearly reduced the DNA fragmentation rate. In conclusion, 10% DMSO can effectively reduce the ultrastructural damage, oxidative damage, and DNA damage of yellow catfish spermatozoa during cryopreservation; it can also further optimize the cryopreservation protocol for yellow catfish spermatozoa. Meanwhile, it also provides a theoretical basis for the future optimization of the cryopreservation protocols.

## 1. Introduction

Yellow catfish (*Pelteobagrus fulvidraco*) is an omnivorous freshwater fish widely distributed in major inland water systems in China. Its tender flesh and the absence of intermuscular spines have made it a commercially important species in China as well as Southeast Asia [1,2]. Under the same feeding conditions, the economic value of males is much higher than that of females. Thus, selective breeding experiments for YY super-male and XY all-male have been carried out on this species [3]. In mass production, the dendritic structure of yellow catfish’s testes makes it difficult to obtain the semen by pressing its abdomen. The only possible means to obtain testes is via dissection, and the testes will be ground. Killing fish to obtain spermatozoa is wasteful. For this reason, spermatozoa cryopreservation is crucial for the artificial fertilization of yellow catfish.

In as early as 1953, Blaxter successfully carried out spermatozoa cryopreservation in Atlantic herring [4]. With the development of fish spermatozoa cryopreservation, the research has addressed the problems about cryoprotectants, types, and concentrations of extenders, freezing rates, and thawing methods [5,6]. The cryopreservation protocols developed at home and abroad for hundreds of aquatic animals can not only synchronize gamete suitability among species but also benefit biodiversity and genetic preservation. So far, spermatozoa cryopreservation has been applied to over 200 kinds of fish [3,7,8]. Studies in catfish have made great achievements, including the best cryopreservation method for Basa fish (*Pangasius bocourti*) [9], the cryopreservation method for blue catfish (*Ictalurus furcatus*) and its use to improve the genetic germplasm of blue fish [10]. spermatozoa cryopreservation has also been applied to South American catfish (*Rhamdia quelen*) [11], African catfish (*Clarias gariepinus*) [12], Amazonian catfish (*Leiarius marmoratus*) [13] and Pabda catfish (*Ompok pabda*) [14] etc. By now, our laboratory has given specific spermatozoa cryopreservation protocols for yellow catfish [1] and found the effects of spermatozoa cryopreservation on the gene expression of yellow catfish [2]. There has been no research to investigate the mechanism of cryo-injury. The research on freezing damage of spermatozoa in yellow catfish is crucial for the development of new spermatozoa freezing preservation protocol, which has important positive significance for the large-scale artificial production of yellow catfish.

Although cryopreservation techniques have been successful in many respects, spermatozoa are inevitably damaged in this process. Their normal function and reproductive performance are disturbed [15]. To date, many studies have found that cryopreservation can cause damage to the morphology and ultrastructure of spermatozoa, including plasma membrane integrity, spermatozoa nuclei, perinuclear membranes, mitochondrial activity, reactive oxygen species production, and location, level, and function of spermatozoa proteins [16]. The major damages are mainly in three aspects: ultrastructural damage, oxidative damage, and DNA damage. The key to a successful spermatozoa cryopreservation is to find the optimal freezing rate. When the freezing rate is high, the intracellular water cannot flow out completely. The remaining water will form crystals, causing cryo-injury [17]. A low freezing rate results in dehydration of the cell as well as shrinkage of the volume of organelles and membrane structures. The solution or electrolyte concentration will lead to cryo-injury [18], and what occurs first is ultrastructural damage to the plasma or organelle membranes, mitochondria, and other organelles. One of the main reasons for damage during low-temperature freezing storage in the yellow catfish is the oxidative stress induced by the increase of reactive oxygen species (ROS) and the imbalance between antioxidant systems during cryopreservation [19,20], and ROS is the main reason for DNA damage in this process [21]. In fact, ROS have been found to affect spermatozoa viability, lipid peroxidation, mitochondrial membrane potential, and DNA integrity in fresh spermatozoa in bulls and buffaloes [22].

During spermatozoa cryopreservation, cryoprotectants can mitigate the damage caused by the formation of intracellular ice crystals and cellular dehydration [23]. There are mainly two kinds of cryoprotectants: non-osmotic cryoprotectants which generally consist of high molecular weight substances. These cryoprotectants can be monosaccharides, disaccharides, or polysaccharides. They can also be polyvinylpyrrolidone (PVP) or hydroxyethyl starch (HES) [24], or proteins from milk, egg yolks, and vegetable oils [25]. These cryoprotectants are macromolecular so that they can increase the osmotic pressure in the extracellular space, dehydrate the cell during freezing, and prevent over-osmotic swelling during thawing [26]. Another type of cryoprotectant is osmotic cryoprotectants which usually have low molecular weights that are uncharged and soluble in water [24]. Common cryoprotectants used in the laboratory are dimethyl sulfoxide (DMSO), dimethylacetamide (DMA), glycerol (Gly), propylene glycol, ethylene glycol (EG), etc. They can penetrate the cell and hydrate with water molecules, increasing the viscosity of the solution to reduce ice crystal formation [27]. After long-term research, it has been found that DMSO is the best cryoprotectant for freshwater fish during cryopreservation [28,29]. Therefore, this paper aims to evaluate the effects of adding or not adding DMSO during low-temperature storage on the ultrastructural damage, oxidative damage, and DNA damage of thawed yellow catfish spermatozoa. It will help us to better understand the mechanism of spermatozoa damage in cryopreservation and to re-optimize spermatozoa cryopreservation protocols for yellow catfish.

## 2. Materials and Methods

### 2.1. Broodfish and Gamete Collection

This experiment began in June 2017, and the naturally matured male individuals of yellow catfish (2–3 years old, 254.6 ± 45.4 g body weight) used in the experiment were provided by Bairing Improved Aquatic Seed Company in Foshan, Guangdong. Under natural photoperiod (14 h of light/10 h of darkness), male fish were kept in indoor water tanks with a stocking density of approximately three individuals/m^3^. During the experiment, the water temperature varied from 23 °C to 28 °C. The fish were fed with a commercial diet at 4–5% of body weight twice a day, but feeding was stopped 24 h before the experiment. Due to the difficulty of producing semen by pressing the abdomen in males, it was necessary to collect testicles through euthanizing male specimens. After removing blood and attached tissues, the testicles were suctioned dry and dissected with a surgical knife, and the semen was released into a sterile culture dish, and then the sample was stored in a refrigerator at 4 °C before use.

All fish treatments in this study were conducted in accordance with the guidelines of the Institutional Animal Care and Use Committee of Sun Yat-sen University.

### 2.2. Spermatozoa Quality Evaluation

We used an Integrated Semen Analysis System (ISAS 2.0, Spain) to analyze spermatozoa motility parameters at room temperature (approximately 25 °C). Sampling was carried out with a 10× negative-phase objective. We set the software parameters in the system with a frame rate of 30 images per second and a head recognition area of 1–90 µm^2^. Spermatozoa with a curve velocity (VCL) greater than 10 µm/s were defined as motile. For motility analysis, the spermatozoa were activated with distilled water (containing 0.1% BSA to avoid spermatozoa viscosity) in a ratio of 1:49, quickly activating the spermatozoa. A 5 µL mixture was immediately (within 5 s) transferred into the 10-µm-depth spermatozoa counting chamber, and more than 500 cells from three different fields were randomly captured by the system. Measurement of motility was repeated in duplicate for each sample. Subsequent experiments will only use fresh spermatozoa with an initial vitality greater than 90%. After dilution by 1000 times, the spermatozoa density was measured using the ISAS software concentration module.

### 2.3. Spermatozoa Cryopreservation and Thawing

In this experiment, spermatozoa were frozen in a Styrofoam box containing liquid nitrogen [1]. Before freezing, the straws and cryomedium were kept on crushed ice. Mixing the semen of three male fish with spermatozoa vitality exceeding 90% together, the volume was 2 mL. We divided it equally into a fresh semen group (without treatment) and a Cryogenic group without a protective agent (DMSO^−^) (diluted with 0.9% NaCl) and added a protective agent (DMSO^+^) to the frozen semen group (diluted with 0.9% NaCl in a ratio of 1:3 and added with 10% DMSO). Each treatment included three parallel experiments, with each parallel experiment tested three times. After loading the sample into a 0.25 mL straw (IMV, L’Aigle, France), we placed it horizontally on a floating plate at a height of 7 cm from the liquid nitrogen surface and equilibrated it for 10 min. After freezing, the pipette should be stored in liquid nitrogen for at least 48 h before assessment. For thawing, the straw was removed from liquid nitrogen and immediately thawed in a 40 °C water bath for 5 s. Then, we dried the straw and cut it open with scissors, allowing the sample to flow into a 1.5 mL centrifuge tube. The thawed sample was temporarily placed on crushed ice (or stored at 4 °C after thawing) and then subjected to dynamic analysis experiments.

### 2.4. Ultrastructure Examination

#### 2.4.1. Scanning Electron Microscope (SEM) Observation

Spermatozoa samples (fresh spermatozoa/DMSO^+^/DMSO^−^) were first shaken and fixed in 2.5% glutaraldehyde fixative then rinsed with PBS (3 times, 10 min each time). Rinsed clean spermatozoa were fixed in 1% osmium solution for another 90 min; we rinsed the fixed spermatozoa with PBS (once, 10 min), and then dehydrated them in a gradient concentration of ethanol solution (30, 50, 70, 80, and 100%; 10 min each time, repeating 100% concentration dehydration once). Finally, we replaced it with Isoamyl Acetate (once at 50% concentration, twice at 100% concentration, with a replacement time of 10 min each time). The sample that had been replaced was placed in a carbon dioxide critical point dryer (XD-1, Eiko, Sakata, Japan) for drying. The dried powder was then placed in an ion plating instrument (IB3, Eiko, Sakata, Japan) for gold plating. The gold-plated sample was placed in a scanning electron microscope (Hitachi S-3400N, Hitachi, Japan), and different areas were selected for observation and photography.

#### 2.4.2. Transmission Electron Microscopy (TEM) Observation

Three groups of samples were washed once each with PBS buffer and then fixed with an equal volume of 2.5% glutaraldehyde fixative. The samples were rinsed three times with PBS (pH = 7.4) buffer for 10 min each time. Subsequently, they were fixed with a 1% citric acid solution at 4 °C for 2 h, rinsed with PBS buffer three times for 10 min each time, and then dehydrated with ethanol concentrations of 30%, 50%, 70%, 80%, and 100% at each gradient concentration for 10 min, with 100% ethanol dehydration repeated once. They were embedded with Epon812 epoxy resin (HEAD, Beijing, China) and cured in temperature chambers of 37 °C, 45 °C, and 65 °C for 24 h at each stage. They were sliced using an UltracuttE, stained with lead acetate uranyl nitrate, and finally observed by transmission electron microscopy (JEM-100CX—Ⅱ JEOL, Tokyo, Japan).

### 2.5. Enzyme Activity Assay

#### 2.5.1. SOD Assay

This experiment used the enzyme activity commercial kit from Nanjing Jiancheng Biotech Co. (Nanjing, China) to analyze the SOD enzyme activity of yellow catfish spermatozoa before and after cooling, and the corresponding operations were carried out according to the instructions. Its principle was that when the SOD content in the spermatozoa sample was low, superoxide anion radicals were oxidized hydroxylamine heavily, and finally, under the action of the color reagent in the detection kit, the product nitrite would appear purple-red. On the contrary, when there was a high amount of SOD in the spermatozoa sample, the superoxide anion radicals would be inhibited, and it would be specific. Eventually, under the action of the color reagent in the detection kit, the formation of nitrite would be reduced, and the color would be weak. Thus, we adjusted the spectrophotometer to a wavelength of 550 nm; used a 1 cm optical path colorimetric cup, zero with double distilled water; tested the absorbance values of each test tube; and compared the absorbance values of the measuring tube with those of the control tubes to calculate the concentration of superoxide dismutase (SOD).

#### 2.5.2. MDA Assay

This experiment used an improved Thiobarbituric Acid Reactive Substances (TBARS) method to detect Malondialdehyde (MDA)levels, which can reflect the amount of lipid peroxides in extracellular fluid, cellular level, and subcellular level after testing. Malondialdehyde (MDA) was a product of lipid peroxidation degradation, and after condensation with Thiobarbituric acid (TBARS), it formed a red product with the highest absorption peak at 532 nm. The difference between the absorbance of the standard tube and the absorbance of the blank tube was within the range of 0.065–0.070, indicating that the standard tube meet the standard. After obtaining the supernatant according to the operation, we used a 1 cm optical path colorimetric cup and doubled distilled water to zero at 532 nm of the spectrophotometer to test the absorbance values of each test tube and calculate the MDA content.

#### 2.5.3. T-AOC Assay

We used the T-AOC detection kit for total antioxidant capacity testing, with ABTS (2,2′-azino bis (3-ethylbenzthiazoline-6-sulfonic acid0) as the color reagent for this kit and performed the testing according to the manufacturer’s protocol. In short, first, we configured the ABTS working fluid, and prepared 80 µL of working solution using 40 µL of ABTS and 40 µL of oxidant each (after stabilizing at room temperature in the dark for 12–16 h, diluted 30–50 times with PBS). The absorbance of the working solution should be subtracted from the blank control to determine the value at 734 nm on the enzyme-linked immunosorbent assay (ELISA) reader, which should be within the range of 0.7 ± 0.05. Approximately 10^6^ spermatozoa samples were collected in centrifuge tubes, adding 200 µL of refrigerated PBS solution, and homogenizing to thoroughly crush cells (4 °C, 12,000 r/5 min). We measured the absorbance of the supernatant at 734 nm and compared it with the absorbance of the TBARS standard curve to calculate the total antioxidant capacity of the sample.

The detection of SOD, MDA, and T-AOC required the determination of protein concentration to obtain the activity units per milligram of protein. In summary, we prepared a working solution for measuring protein content using BCA solvents A and B in a 50:1 ratio. It was sealed and stored at room temperature for 24 h to stabilize. The 2 mg/mL BSA standard was diluted with PBS into solutions with gradients of 0, 125, 250, 500, 750, 1000, 1500, and 2000 µg/mL. We added 200 µL of working solution to each well of the enzyme-linked immunosorbent assay (ELISA) plate detection well and then used a pipette to draw 25 µL of gradient standard solution and 25 µL of spermatozoa sample each into the detection well at a ratio of Sample/WR = 1:8. After mixing, the vibrator vibrated for 30 s. We covered the plate and incubated in a 37 °C constant temperature incubator for 30 min. After removing the constant temperature box, cooling it to room temperature and measuring the absorbance at 562 nm using an enzyme-linked immunosorbent assay (ELISA) reader.

### 2.6. DNA Integrity Testing

**Figure 1:**
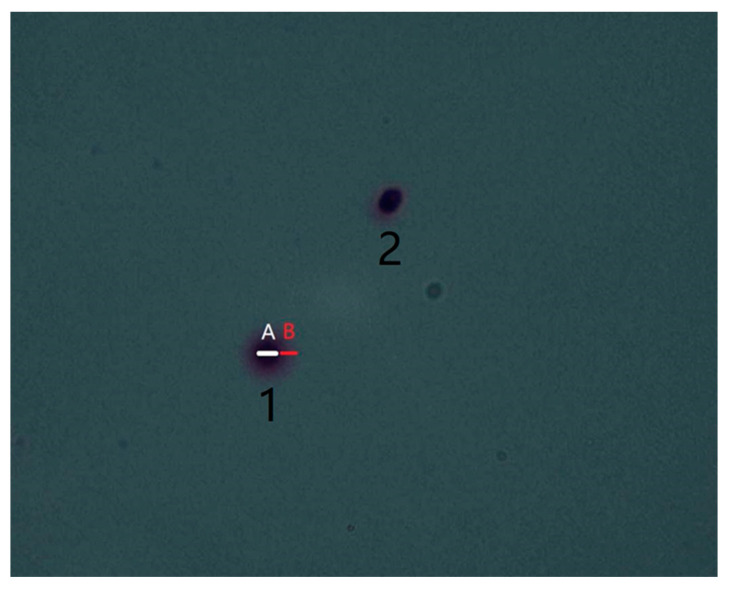
Criteria for determining DNA fragments of spermatozoa. A: the minimum diameter of the spermatozoa head. B: width of unilateral halo. 1: Spermatozoa with intact DNA; 2: Spermatozoa with DNA fragments.

This experiment used the spermatozoa Chromatin Diffusion (SCD) method to detect DNA damage. Briefly, it was used to melt the fusible gel tube completely in a water bath (80 °C, 20 min) and diluted fresh or frozen semen with PBS to 1–10 × 10^6^/mL before finally adding the sample. At room temperature, we immersed the glass slides vertically in reaction solution A (7 min), reaction solution B (accurate reaction at 20–28 °C for 25 min), purified water (5 min), and ethanol reaction tanks (70%, 90%, 100%, 2 min each time). Firstly, we let it dry naturally in the air then dyed it with Swiss dye, and finally dried it by airing. The spermatozoa labeled as 1 have complete DNA, while the spermatozoa labeled as 2 have DNA fragments. When B ≤ A, it can be confirmed that the spermatozoa have DNA fragments (Figure 1). We observed at least 200 spermatozoa and counted those with DNA fragments present. The spermatozoa DNA fragmentation rate (%) is the ratio of the number of spermatozoa with DNA fragments to the total number of observed spermatozoa.

### 2.7. Statistical Analysis

All results were expressed as mean ± SD. We used SPSS 20.0 software (SPSS, Chicago, IL, USA) to perform statistical analyses. The percentage data were transformed by arcsine square root. We used the Shapiro–Wilk test to check whether the data fit the normal distribution. We used the one-way ANOVA and Duncan’s multiple-range tests. If the data did not conform to normality, the Kruskal–Wallis test was used for non-parametric testing. Pearson’s correlation test was used to check the correlation between the parameters, with a significance level of *p* < 0.05.

## 3. Results

### 3.1. Measurement of Movement Parameters

According to Table 1, it can be observed that the sperm motility of the DMSO^+^ group (75.64 ± 1.12) is significantly lower than that of the fresh sperm group (95.84 ± 0.36) but significantly higher than that of the DMSO^−^ group (48.99 ± 4.46) (*p* < 0.05); the VCL and VSL parameters of sperm in the DMSO^+^ group are significantly lower than those in the fresh sperm group, but compared with the DMSO^−^ group, both VCL and VSL are significantly increased (*p* < 0.05).

### 3.2. Ultrastructure Analysis

**Figure 2:**
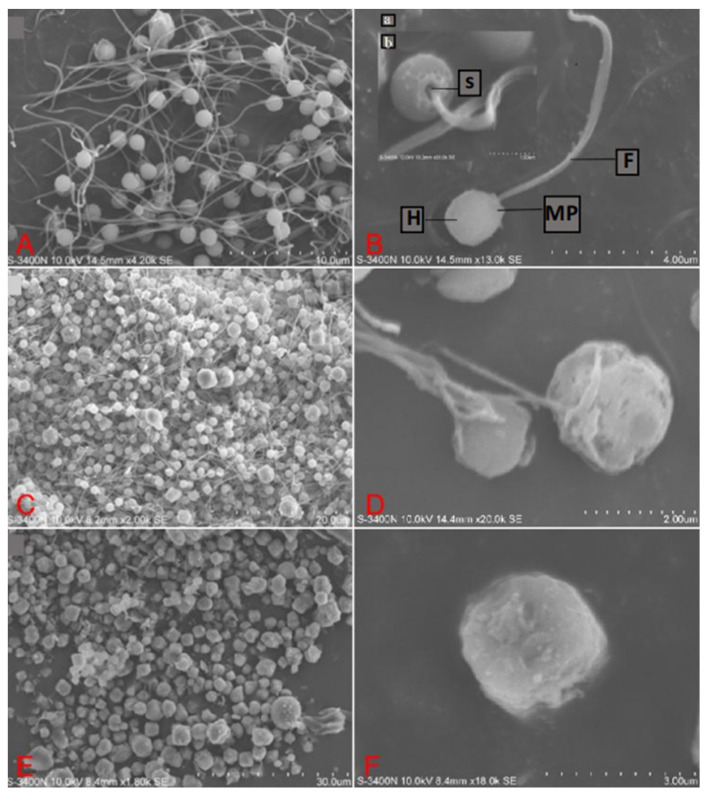
Scanning electron micrographs of fresh and post-thaw spermatozoa. (**A**,**B**) Fresh spermatozoa images under scanning electron microscopy (H: head; MP: midpiece of spermatozoa; F: tail; S: central space of sleeve; a: Whole spermatozoa scanning electron microscopy; b: Localized scanning electron microscopy of spermatozoa); (**C**,**D**) the images of thawed spermatozoa stored in 10% DMSO under scanning electron microscopy; (**E**,**F**) images of frozen spermatozoa under scanning electron microscopy.

Spermatozoa scanning electron microscopy shows that, overall, most fresh spermatozoa have smooth surfaces and intact structures, with a few experiencing swelling or damage to the head, flagella breakage, or detachment due to experimental procedures (Figure 2A). Spermatozoa are composed of three parts: the head, midpiece, and the flagella. The head is round, while the midpiece is concave and connected to a slender and smooth flagellum (Figure 2B). After thawing the spermatozoa stored in 10% DMSO, it can be observed that the majority of spermatozoa still retain their intact structure, but the phenomenons of swollen or ruptured spermatozoa heads and broken flagella increased (Figure 2C). However, the head of the spermatozoa was still spherical, but the surface was not as smooth as fresh spermatozoa, and there was even some swelling and rupture. The middle section showed some degree of damage, and the number of flagellar breaks increased. (Figure 2D). Unprotected spermatozoa showed almost no intact cells as a whole, resulting in extensive damage (Figure 2E) and even severe deformities of the sperm head, midsection breakage, and flagella detachment (Figure 2F).

**Figure 3:**
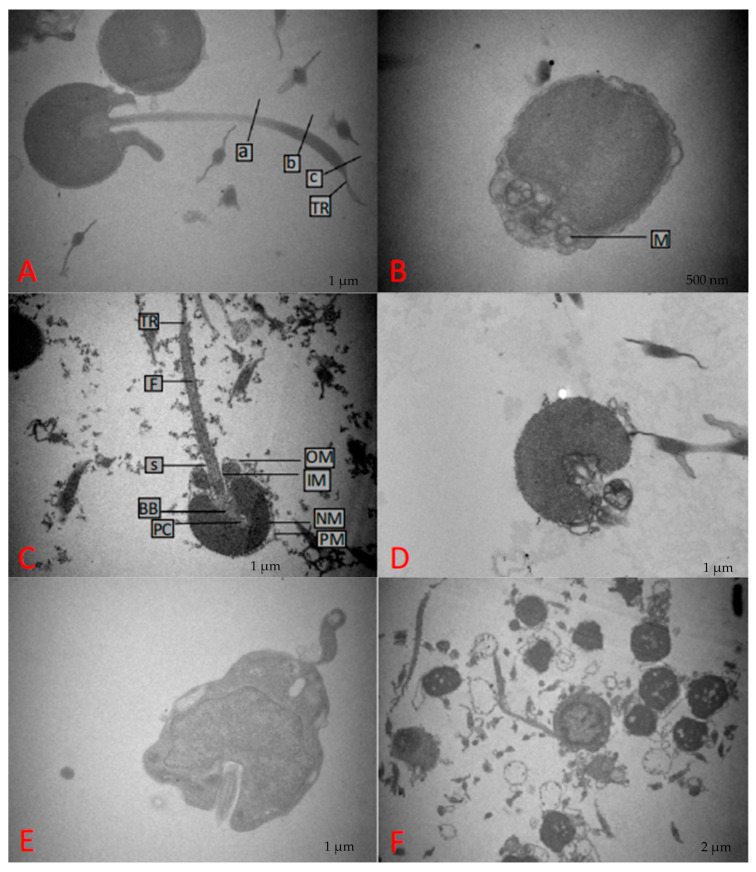
Transmission electron micrographs of fresh and post-thaw spermatozoa. (**A**,**B**) Images of fresh spermatozoa under transmission electron microscopy (a, b, c: microtubule structures; M: mitochondria; (**C**,**D**) images of thawed spermatozoa stored in 10% DMSO under transmission electron microscopy (PM: plasma membrane; NM: nuclear membrane; PC: proximal centriole; BB: basal baby; OM: outer membrane of sleeve cover; IM: inner membrane of the sleeves; S: central space of the sleeve; F: flagellum; TR: transition region); (**E**,**F**) images of frozen spermatozoa under transmission electron microscopy.

Spermatozoa transmission electron microscopy shows that fresh spermatozoa from yellow catfish have a typical spherical head without an acrosome structure. The nucleus accounts for a very large proportion and is tightly attached to the nuclear membrane, while the cytoplasm accounts for a very small proportion. The middle section is concave inward toward the head, with two mitochondria wrapped in sleeve membranes next to it. The middle section is connected to slender flagella below, and the majority of the cell structures are intact without obvious damage (Figure 3A). However, flagellar loss and slight swelling of the plasma membrane were observed (Figure 3B). After thawing in 10% DMSO, most of the spermatozoa retained their intact structure, while some showed slight damage such as mitochondrial swelling and membrane wrinkling (Figure 3C), and others showed membrane rupture, mitochondrial loss, and flagellar detachment (Figure 3D). Unprotected spermatozoa showed severe cellular abnormalities, plasma membrane shedding, mitochondrial loss, and flagellar loss (Figure 3E). Overall, they were mostly irregular in shape and there were hardly any cells similar to fresh spermatozoa. They were in a state of apoptosis and death (Figure 3F).

### 3.3. Enzyme Activity Analysis

The analysis of spermatozoa enzyme activity in yellow catfish showed that although the SOD enzyme activity in the DMSO^+^ group (7.99 × 10^4^ ± 5.24 nmol/mgprot) was significantly lower than that in the fresh spermatozoa group (1.45 × 10^5^ ± 3.10 nmol/mgprot), it was significantly higher than that in the DMSO^−^ group (4.56 × 10^4^ ± 1.17 nmol/mgprot) (*p* < 0.05); the SOD enzyme activity in the DMSO^−^ group was significantly lower than that in the fresh spermatozoa group (*p* < 0.05). The MDA activity in both the fresh spermatozoa group and the DMSO^+^ group was very low, with no significant difference between them (*p* > 0.05), but they were significantly lower than the DMSO^−^ group (7.03 ± 0.32 nmol/mgprot) (*p* < 0.05). The T-AOC enzyme activity in the fresh semen group and DMSO^+^ group were 4.1 × 10^5^ ± 0.03 and 3.7 × 10^5^ ± 0.06 nmol/mgprot, respectively, with no significant difference between the two (*p* > 0.05), but both were significantly higher than that in the DMSO^−^ group (8.0 × 10^4^ ± 0.02 nmol/mgprot) (*p* < 0.05).

### 3.4. DNA Integrity

**Figure 4:**
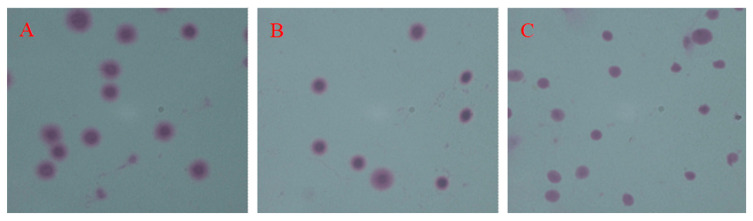
DNA fragments of fresh spermatozoa, DMSO^+^, and DMSO^−^ groups of yellow catfish spermatozoa under 100× oil microscope. (**A**) DNA fragments in fresh spermatozoa; (**B**) DNA fragments in the DMSO^+^ group; (**C**) DNA fragments in the DMSO^−^ group.

**Figure 5:**
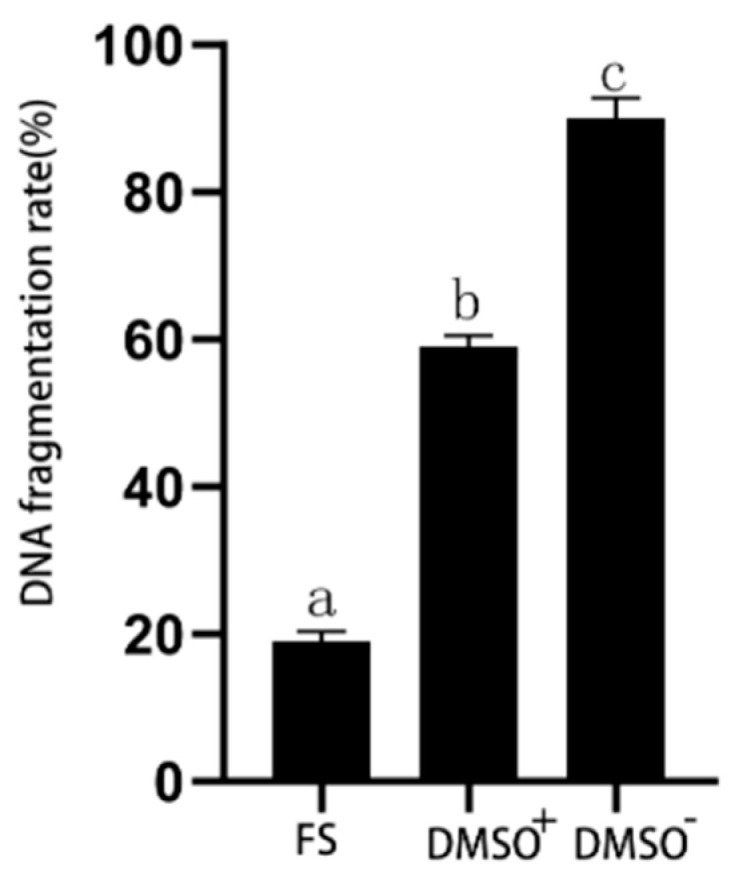
DNA fragmentation rate of fresh spermatozoa, DMSO^+^, and DMSO^−^ groups of yellow catfish spermatozoa (%). FS: fresh spermatozoa; different superscripts refer to significant differences (*p* < 0.05).

Chromatin diffusion analysis showed that the spermatozoa DNA fragmentation rate in the DMSO^+^ group (59 ± 1.56%) was significantly higher than that in the fresh spermatozoa group (19 ± 1.34%) but significantly lower than that in the DMSO^−^ group (90 ± 2.77%) (*p* < 0.05). The DNA fragmentation rate in the DMSO^−^ group was significantly higher than that in the fresh semen group (*p* < 0.05) (Figure 4 and Figure 5).

## 4. Discussion

DMSO is a small amphipathic molecule with two nonpolar methyl groups and a polar sulfoxide group. Such a structure allows it to easily cross cell membranes and effectively prevent the formation of intracellular ice crystals, eventually mitigating cryoinjury [30]. It may even reduce osmotic stress and cellular dehydration [31], providing protection for spermatozoa. This study shows that the addition of 10% DMSO to spermatozoa not only significantly improves sperm motility compared to spermatozoa without protective agents but also greatly attenuates the ultrastructural damage, oxidative damage, and DNA damage caused by the cryopreservation of yellow catfish spermatozoa.

The ultrastructure is an important criterion for evaluating the quality of spermatozoa during cryopreservation. Damage to the structural integrity of spermatozoa can be observed in mitochondria, chromatin, or membranes [32]. Studies on spermatozoa cryopreservation in 10% DMSO of Cobia (Rachycentron canadum) have found that compared with fresh spermatozoa, cryo-preserved spermatozoa have head-swelling and surface irregularities. The transmission electron microscope also revealed rupture and detachment of the plasma membranes [33]. When the spermatozoa of the spotted fish were cryo-preserved with a combination cryoprotectant of 5% DMSO and 5% EG, the surface of the spermatozoa remained smooth under the scanning electron microscope, but their heads swelled, and the plasma membrane contracted. The organelle structure was basically intact, but a few cells had their plasma membranes ruptured, mitochondria lost, and flagellum disappeared [34,35]. This experiment observed similar phenomena. For spermatozoa cryopreserved with 10% DMSO, most of them had intact organelle structures. Only a few cells had their plasma membranes wrinkled or broken, their mitochondria swollen or lost, and their flagellum ruptured or disappeared. Meanwhile, most of the spermatozoa in the DMSO^−^ group underwent severe malformations, with dissolution or even disappearance of the membrane structure. The spermatozoa were overall in a dead state. This proved that 10% DMSO can mitigate the structural damage to spermatozoa caused by cryopreservation. The explanation may be that DMSO can interact with the polar head groups of phospholipids within the phospholipid bilayer. The interaction guarantees the structural stability of the membrane at a certain concentration, which mitigates the damage to the spermatozoa membrane structure caused by freeze–thawing [35]. In addition, it was found that when cryopreserving the spermatozoa of black bass, 10% DMSO provides the best protection for their plasma membrane [36]. The plasma membrane receives major damage from warming and cooling [37]. This damage to the membrane structure affects its permeability and the transport of enzymes on the membrane surface. Then, spermatozoa become more susceptible to damage during freezing or thawing [38]. Therefore, future optimization can focus on mitigating plasma membrane damage to better protect the spermatozoa of yellow catfish and improve their quality.

Reactive oxygen species (ROS) are considered to be one of the main causes of impaired integrity and impaired function of fish spermatozoa during cryopreservation [39]. spermatozoa cells are vulnerable to ROS attack because they have a high level of unsaturated fatty acids and the antioxidant capacity of diluted semen is reduced [40,41]. Superoxide dismutase (SOD) is an important antioxidant enzyme. It can catalyze the disproportionation of free superoxide anion radicals to hydrogen peroxide, which is subsequently converted to water and oxygen via catalase or glutathione peroxidase [42]. Scholars compared four families of bony fish (Salmonidae, Salmonidae, Percoidei, and Cyprinidae). Their analysis shows that the enzymatic antioxidant defense system of seminal plasma and spermatozoa of bony fish consists of SOD, CAT, peroxidase, glutathione reductase, and methionine reductase. Activities of all enzymes, except SOD, are very low [43]. This proves the importance of SOD in the antioxidant system. Moreover, one of the major results of oxidative damage in spermatozoa is an increase in lipid peroxidation [44,45,46]. Malondialdehyde (MDA) is a secondary product of lipid peroxidation, and the level of MDA reflects the degree of lipid peroxidation [47]. The total antioxidant capacity (T-AOC) reflects the level of cellular antioxidants in general. This experiment found that compared with frozen spermatozoa, the activities of SOD, MDA, and T-AOC enzymes of cryopreserved spermatozoa with 10% DMSO were significantly increased (Table 2). This indicated that the antioxidant capacity was clearly improved, and the lipid peroxidation damage was also largely reduced, which was proven by the improvement of the plasma membrane structure in ultrastructure with 10% DMSO (Table 2). The total antioxidant capacity of cryopreserved spermatozoa was even comparable with that of the fresh spermatozoa, which meant that the addition of 10% DMSO could effectively reduce the oxidative damage to spermatozoa of yellow catfish. Firstly, the author of this paper suggests that an appropriate concentration of DMSO can reduce the damage of cryopreservation to spermatozoa organelles and membrane structure, reduce the leakage of antioxidant enzymes, and stabilize the antioxidant defense system. It will further strengthen the resistance to ROS and alleviate oxidative damage. Secondly, DMSO has strong permeability and contains sulfur atoms in its chemical structure. The lone pair electrons on the sulfur atoms can allow DMSO to participate in certain free radical reactions, thereby reducing oxidative stress damage. Previous studies have shown that oxidative damage alters the assembly, composition, and kinetic structure of plasma membranes [47,48], and it will ultimately alter their fluidity and permeability [49] and enzymatic activity [50]. Thus, oxidative stress brings enormous damage to spermatozoa during cryopreservation. The future cryopreservation protocol of yellow catfish spermatozoa can be optimized in terms of reducing ROS production and mitigating oxidative stress.

DNA integrity is one of the most important indicators for assessing spermatozoa quality [51,52]. The damage to DNA affects the genetic stability of the offspring, which directly affects the rates of fertilization rate, embryo development, and its survival [53]. Therefore, it is necessary to assess DNA integrity. A number of scholars have explored the causes of DNA damage during cryopreservation. Rani and Munuswamy suggest that when cryopreserving batfish semen, DNA damage is mainly caused by the formation of ice crystals which brings mechanical damage. The DNA damage may also be related to the toxicity of cryoprotectants [54]. Results of integrity testing of spermatozoa DNA in a large yellow croaker (*Pseudosciaena crocea*) show that the toxicity of cryoprotectants is the main cause of DNA damage in spermatozoa [55]. Some scholars have also suggested that spermatozoa DNA damage during cryopreservation was mainly caused by the production of reactive oxygen species (ROS) [56]. ROS attack the DNA at the sugar level, leading to its fragmentation, base loss, or strand breaks [57]. Therefore, factors including mechanical damage during operation, ice crystal damage, cryoprotectant toxicity, and oxidative stress may cause DNA damage and lead to a decrease in spermatozoa quality. Scholars also investigated the effects of cryopreservation on mitochondria. Irvine et al. concluded that spermatozoa with high levels of DNA damage generally had a lower rate of motility, suggesting that the DNA damaged by cryopreservation was likely to be the DNA associated with mitochondrial activity [58]. Mitochondria are organelles with a double membrane and their own DNA. Mitochondria also have multiple copies. When cryopreservation causes damage to fish spermatozoa, their mitochondria are subject to alterations in the structural function and genetic mechanisms of mitochondria [59]. In this experiment, the DNA fragmentation rate was significantly reduced when adding the cryoprotectant compared to the DMSO^−^ group. It indicates that 10% DMSO can protect DNA to a certain extent. For the specific mechanism, firstly, we believe that adding DMSO at an appropriate concentration reduces the damage of ice crystals to the plasma membrane, nuclear membrane, and mitochondrial inner membrane, or the dehydration shrinkage caused by rapid dehydration, thereby protecting DNA. In addition, we believe that after DMSO penetrates cells, it reacts with free radicals, reducing the attack of ROS on DNA and thus reducing the degree of DNA damage.

## 5. Conclusions

This experimental study found that cryopreservation not only causes a decrease in spermatozoa motility and ultrastructural damage in yellow catfish but also generates ROS, leading to oxidative damage and even affecting DNA. Using 10% DMSO as a cryoprotectant can improve spermatozoa motility (75.64 ± 1.12), significantly alleviate injuries to organelles and membrane structures, improve SOD (7.99 × 10^4^ ± 5.24 nmol/mgprot) and T-AOC (3.7 × 10^5^ ± 0.06 nmol/mgprot) enzyme activity, reduce MDA (1.09 ± 0.45 nmol/mgprot) enzyme activity, and mitigate the degree of oxidative damage. It also greatly reduced the DNA fragmentation rate (59 ± 1.56%). The research results provide data support for the optimization of the cryopreservation protocol for the spermatozoa of yellow catfish, which is beneficial for the preservation of the germplasm resources of yellow catfish and the effective utilization of resources during large-scale production. At the same time, it also provides direction for the selection of cryoprotectants for the preservation of other fish germplasm resources in the future.

## Figures and Tables

**Table 1:** Comparison of sperm motility and movement among fresh spermatozoa group, DMSO^+^ group, and DMSO^−^ group of yellow catfish.

Group	Motility (%)	VCL (μm/s)	VSL (μm/s)
Fresh spermatozoa	95.84 ± 0.36 ^a^	114.74 ± 2.45 ^a^	65.17 ± 6.19 ^a^
DMSO^+^	75.64 ± 1.12 ^b^	50.13 ± 3.33 ^b^	20.33 ± 1.34 ^b^
DMSO^−^	48.99 ± 4.46 ^c^	31.40 ± 1.69 ^c^	13.27 ± 0.27 ^b^

Values with different superscript letters are significantly different (*p* < 0.05). VCL: curve line velocity; VSL: straight line velocity.

**Table 2:** Superoxide dismutase (SOD), malondialdehyde (MDA), and total antioxidant capacity (T-AOC) levels of fresh spermatozoa, DMSO^+^, and DMSO^−^ groups of yellow catfish spermatozoa.

Group	SOD (nmol/mgprot)	MDA (nmol/mgprot)	T-AOC (nmol/mgprot)
Fresh spermatozoa	1.45 × 10^5^ ± 3.10 ^a^	0.89 ± 0.11 ^a^	4.1 × 10^5^ ± 0.03 ^a^
DMSO^+^	7.99 × 10^4^ ± 5.24 ^b^	1.09 ± 0.45 ^a^	3.7 × 10^5^ ± 0.06 ^a^
DMSO^−^	4.56 × 10^4^ ± 1.17 ^c^	7.03 ± 0.32 ^b^	8.0 × 10^4^ ± 0.02 ^b^

Values with different superscript letters are significantly different (*p* < 0.05).

## Data Availability

Data are contained within the article. Informed consent was obtained from all subjects involved in the study.
